# Advance Care Planning in the Context of Dementia: Defining Concordance

**DOI:** 10.1093/geront/gnae029

**Published:** 2024-03-27

**Authors:** Jordana L Clayton, Katherine P Supiano, Nancy Aruscavage, Sara G Bybee, Rebecca L Utz, Eli Iacob, Kara B Dassel

**Affiliations:** Gerontology Interdisciplinary Program, College of Nursing, University of Utah, Salt Lake City, Utah, USA; Gerontology Interdisciplinary Program, College of Nursing, University of Utah, Salt Lake City, Utah, USA; Gerontology Interdisciplinary Program, College of Nursing, University of Utah, Salt Lake City, Utah, USA; Gerontology Interdisciplinary Program, College of Nursing, University of Utah, Salt Lake City, Utah, USA; Gerontology Interdisciplinary Program, College of Nursing, University of Utah, Salt Lake City, Utah, USA; Gerontology Interdisciplinary Program, College of Nursing, University of Utah, Salt Lake City, Utah, USA; Gerontology Interdisciplinary Program, College of Nursing, University of Utah, Salt Lake City, Utah, USA

**Keywords:** Decision making, End-of-life care, Qualitative research methods, Surrogate decision making

## Abstract

**Background and Objectives:**

Individuals with dementia may require a surrogate decision maker as their disease progresses. To prepare for this potential role, dementia care partners need to develop a thorough understanding of their care recipient’s end-of-life values and preferences, or care dyad advance care planning (ACP) concordance. As part of our pilot study implementing the LEAD intervention with dementia care dyads, we conducted a multimethod investigation to define care dyad ACP concordance.

**Research Design and Methods:**

We conducted a scoping review of peer-reviewed studies published after 1991 in English focusing on care dyad ACP concordance in dementia care and included 34 articles. Concurrently, we used descriptive qualitative analysis to analyze 7 dyadic ACP conversations from a pilot study about dyadic dementia ACP.

**Results:**

The scoping review demonstrated (a) no definition of care dyad ACP concordance was reported; (b) surrogate accuracy in end-of-life decisions varies widely; and (c) best practices for ACP in dementia may aid in achieving ACP concordance, but do not prioritize it as an outcome. Qualitative analysis identified 7 elements for achieving concordance: Respect/Regard; use of Clarifying Processes; Conveying Health Care Scenarios; Affirmation of Understanding; Recognizing Uncertainty; Expression of Positive Emotions; and Trust.

**Discussion and Implications:**

Care dyad ACP concordance occurs *when care recipients and care partners both understand a care recipient’s end-of-life values, understand the end-of-life preferences informed by those values, and the care partner expresses a willingness to accomplish the care recipient’s wishes to the best of their ability*. ACP concordance can be further operationalized for research and clinical care.

## Background and Objectives

When individuals are unable to make medical decisions independently, they require a surrogate decision maker to act on their behalf. In the case of dementia, the need for a surrogate decision maker may be inevitable, as the disease involves a decline in cognitive functioning that may make the person with dementia no longer able to understand care options, make independent decisions, or communicate those decisions to their care team. (This paper uses the term dementia to refer to Alzheimer’s disease and all other types of dementia.) In fact, research demonstrates that among hospitalized older adults, a surrogate decision maker is needed at least half of the time and that for older adults who had at least one decision made by a surrogate, 57.2% of these decisions revolved around life-sustaining care (Torke et al., 2014). When unprepared to make such decisions, surrogate decision makers can experience uncertainty, strain, depression, anxiety, and trauma-related symptoms (Anderson et al., 2009; Azoulay et al., 2005; Fetherstonhaugh et al., 2017, 2019; Makino et al., 2020) and care recipients can experience undesired healthcare outcomes (Wilkins, 2021) including artificial nutrition, ventilation, discharge to extended-care facilities, and even hospital mortality (Torke et al., 2014), illustrating the importance of preparing surrogate decision makers through advance care planning (ACP).

The purpose of ACP is to help prepare individuals and their surrogates for making end-of-life care decisions. For care recipients, the ACP process is a time to consider what their preferences and values are for care at the end of life. ACP should also prepare surrogate decision makers should their care recipient become unable to communicate their wishes (Rietjens et al., 2017). Although legal documents like advance directives and do-not-resuscitate forms are commonly considered the basis of ACP, they do not cover many situations that arise in the context of dementia and therefore may not fully prepare surrogate decision makers of people with dementia. For surrogate decision makers to be fully prepared for their role, the dyad needs to develop care dyad ACP concordance, a mutual understanding within the dyad of the care recipient’s ACP values and preferences, and a commitment by the surrogate decision maker to carry out these values and preferences to the best of their ability. Care dyad ACP concordance does not equate to the surrogate decision maker agreeing with the values and preferences of the care recipient; in fact, surrogate decision makers may strongly disagree with or dislike their care recipient’s end-of-life values and preferences. Rather, care dyad ACP concordance refers to the mutual understanding of the care recipient’s wishes for their end-of-life care and a commitment that the surrogate decision maker will adhere to the preferences and values of the care recipient when tasked with making future decisions.

Our team carried out a multimethod investigation with the goal of defining care dyad ACP concordance. First, we carried out a scoping review of the literature. The scoping review was most effective in establishing the qualities and steps care dyads use to achieve ACP concordance and current best practices for dementia-focused ACP. These findings, while important, are not sufficient in describing the full process or outcomes associated with achieving ACP concordance within dyads. In particular, the scoping review emphasized the importance of having informal dyadic ACP conversations, but it did not describe what those conversations look like in practice. Thus, we paired the scoping review with a qualitative analysis of recorded ACP conversations among dementia care dyads that resulted in achieving ACP concordance. Analyzing these conversations provided crucial information on *how* dyads reached ACP concordance. Combining the results of the scoping review with the qualitative analysis allowed us to conceptualize both the process and outcomes of care dyad ACP concordance. This work addresses a gap in the literature by defining a concept frequently employed but rarely examined directly.

## Part 1: Scoping Review

### Scoping Review Background and Objectives

The objectives for the scoping review were to (a) determine if there is an established definition for care dyad ACP concordance, (b) identify measures of care dyad ACP concordance, and (c) ascertain best practices facilitating care dyad ACP concordance. Achieving these objectives will help establish the characteristics and practices of dementia care dyads that aid in ACP concordance and inform our formation of a definition for care dyad ACP concordance. Our team chose to use a scoping review methodology, rather than a systematic review or meta-analysis, because the primary objective of the scoping review was to search for a definition of care dyad ACP concordance in the literature and to ascertain the current breadth of research on this concept.

### Scoping Review Research Design and Methods

#### Concordance synonyms

We considered several synonyms for concordance that are frequently used in the literature. The primary synonyms for “mutual understanding of patient wishes” include concordance, congruence, agreement, proxy agreement, proxy accuracy, proxy knowledge, surrogate agreement, surrogate accuracy, and dyadic agreement. Other synonyms, such as consensus, concurrence, and understanding, were infrequently used. The term “congruence” deserves special mention as it is the most used alternative to concordance. It is also frequently used in the negative: incongruence or incongruent. Due to the frequent use of the term congruence in many fields of study within healthcare, such as symptom appraisal, the study team concluded that the term concordance was most appropriate in describing the mutual understanding of the end-of-life values and preferences of care recipients and their care partners. To further distinguish the meaning of concordance to which we refer, we chose to label the concept “care dyad ACP concordance.”

#### Data sources and searches

Our team followed the steps set forward by Levac et al. (2010) on conducting a scoping review: (a) clarify the research question; (b) create a balance between feasibility and comprehensiveness of the scope; (c) use an iterative, team-based approach to selecting studies; (d) extract data; and (e) use both numerical and qualitative thematic analysis. We searched PubMed, Scopus, Embase, CINAHL, and Ovid through January 23, 2023, for studies published in English on establishing ACP concordance between surrogate decision makers and individuals with dementia. Because the terms “concordance” and “congruence” are both widely used outside of the context of ACP and dementia, these terms returned a large majority of results focused on illness or illness symptom appraisal and other forms of dyadic agreement. Therefore, although we used concordance and congruence as search terms, the terms “proxy” and “surrogate” were more effective in returning results on care dyad ACP concordance. We have included a table summarizing the search strategy in Supplementary Material. Additionally, we performed forward and backward citation chaining to identify additional relevant articles.

Studies were eligible if they (a) were primarily on the topic of ACP; (b) were included or focused on ACP concordance between surrogate decision makers and individuals with dementia; (c) were peer reviewed; (d) were published in English; and (e6) were published after the passing of the 1991 Patient Self Determination Act (PSDA) and before January 1, 2023. The purpose of the PSDA was to ensure a patient’s right to self-determination by increasing the use of ACP documentation in end-of-life care. The passage of this act was a watershed moment in the field of ACP (Nadel et al., 1995), making it a useful starting point for our research.

Studies were considered to focus on ACP concordance if they studied the level of understanding that surrogate decision makers have of their care recipient’s end-of-life values and preferences, the ways that surrogate decision makers form an understanding of those values and preferences, or qualities of care dyads in which the surrogate decision maker has a high-quality understanding of their care recipient’s values and preferences. Because surrogates must first understand what their care recipient wants before they can commit to carrying it out, studying whether and how surrogate decision makers understand the wishes of their care recipient is central to ACP concordance. We used the following definition of ACP which was developed by Rietiens et al. (2017) through a large international Delphi panel:

Advance care planning enables individuals who have decisional capacity to identify their values, to reflect upon the meanings and consequences of serious illness scenarios, to define goals and preferences for future medical treatment and care, and to discuss these with family and health-care providers. ACP addresses individuals’ concerns across the physical, psychological, social, and spiritual domains. It encourages individuals to identify a personal representative and to record and regularly review any preferences, so that their preferences can be taken into account should they, at some point, be unable to make their own decisions. (p.e546)

This definition emphasizes the role of the care recipient in understanding their own end-of-life values and preferences across all care domains and the role of the surrogate decision maker who can understand, record, and carry out the care recipient’s preferences if they become unable to make independent end-of-life care choices. These two features are foundational to care dyad ACP concordance.

The first three authors determined the objectives, search methods, and inclusion and exclusion criteria, and these were approved by the entire research team. The literature search was performed by the first author, who compiled a list of articles that were potentially relevant. The first three authors then chose the relevant studies from that list. Finally, all members of our team reviewed and finalized the list of included articles.

#### Study selection

**Figure 1.**
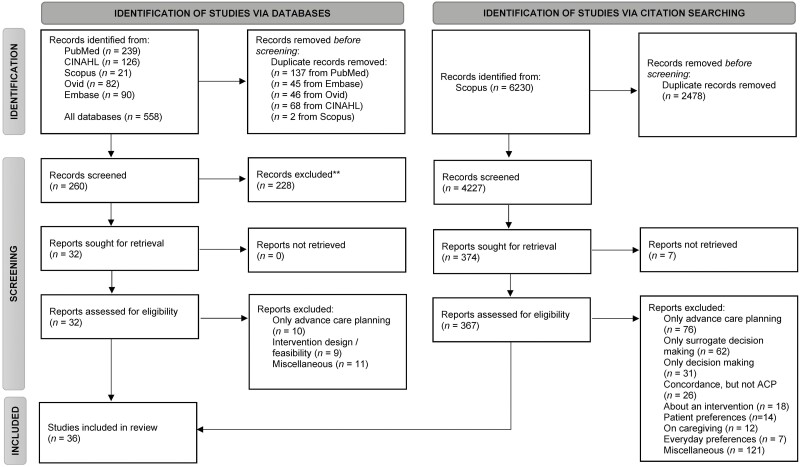
PRISMA flow chart of included studies. From Page MJ, McKenzie JE, Bossuyt PM, Boutron I, Hoffmann TC, Mulrow CD, et al. The PRISMA 2020 statement: an updated guideline for reporting systematic reviews. BMJ 2021;372:n71. doi: 10.1136/bmj.n71. For more information, visit: http://www.prisma-statement.org

As shown in Figure 1, the systematic database search identified 558 articles; citation chaining identified 6,230 articles. We screened articles first by title and excluded articles at this stage only if it was clear from the title alone that the paper would not be relevant. This step resulted in screening 399 article abstracts. We then performed a full-text evaluation on articles that were either eligible or potentially eligible based on the abstract. Based on the inclusion criteria above, we included 34 total papers.

### Scoping Review Results

#### Identified studies

Table 1 summarizes the primary objective and publication date of the 34 included articles. Three (8.8%) articles tested interventions aimed at improving dementia care dyad ACP concordance (Matheis-Kraft & Roberto, 1997; Towsley et al. 2022; Volandes et al. 2009). Two (5.8%) articles tested whether surrogate decision makers were more accurate than a written advance directive (Bravo et al., 2018) or an actuarial model (Smucker et al., 2000). One (2.9%) intervention tested whether older adults with dementia could effectively communicate their end-of-life wishes (Song et al., 2019). Almost all articles were published in the United States; the exceptions are two in Canada (Bravo et al., 2017, 2018) and one each in Hong Kong (Chau et al., 2010), Israel (Ayalon et al., 2012), New Zealand (de Vries & Drury-Ruddlesden, 2019), Switzerland (Bosisio et al., 2018), and the United Kingdom (Dening et al., 2016).

**Table 1.** Distribution of Articles Across Subject and Publication Date

Decade of publication	Surrogate predictive accuracy	Guides for ACP in dementia	Achieving concordance through ACP
1990–1999	Sulmasy et al., 1994 Matheis-Kraft and Roberto, 1997 Sulmasy et al., 1998		
2000–2009	Smucker et al., 2000 Carpenter et al., 2007 Moorman and Carr, 2008 Zettel-Watson et al., 2008 Moorman et al., 2009 Volandes et al., 2009 Whitlatch et al., 2009	Mezey et al., 2000	Black et al., 2009
2010–2019	Chau et al., 2010 Schmid et al., 2010 Parks et al., 2011 Reamy et al., 2011 Turan et al., 2011 Ayalon et al., 2012 Reamy et al., 2013 Dening et al., 2016 Kwak et al., 2016 Bravo et al., 2017 Fried et al., 2017 Bravo et al., 2018 Miller et al., 2018 Miller et al., 2019 Song et al., 2019	Whitlatch, 2013 Bosisio et al., 2018 deLima Thomas et al., 2018 Dassel et al., 2019	de Vries and Drury-Ruddlesden, 2019
2020–2022	Towsley et al., 2022	Macchi and Lum, 2022	

*Notes*: ACP, advance care planning.

#### Objective 1: Determine if there is an established definition for care dyad ACP concordance

The scoping review found no articles with a definition of care dyad ACP concordance that extended beyond the scope of their own study. Many of the articles that examined the ability of surrogate decision makers to predict care recipient preferences did employ a definition of concordance for the purpose of their analysis, such as in Towsley et al. (2022), “Concordance occurred when the coded preferences in the resident’s video and staff and family member response matched” (p. 882). These types of definitions are useful for understanding study results but are not generalizable to all care dyads.

#### Objective 2: Identify measures of care dyad ACP concordance

In completing our second objective, identifying measures of care dyad ACP concordance, most of the articles (26, 76%) examined the ability of surrogate decision makers to accurately predict the end-of-life care preferences and values of their care recipient. All used quantitative methods, and all but one (Kwak et al., 2016) elicited patient preferences using case vignettes or a structured questionnaire. Surrogates completed the questionnaire as if they were deciding for the patient, and then researchers compared patient preferences with the surrogates’ predictions. Kwak et al. (2016) analyzed the relationship between surrogates’ self-reported knowledge of patient values and preferences. Table 2 summarizes these articles.

**Table 2.** Surrogate Decision Makers’ Ability to Predict Patient End-of-Life Preferences Across Disease States

First author	Year	Measure of preferences or values^a^	Number of disease states and type of CI	Method of statistical analysis	Dyads	Surrogate decision-maker accuracy in predictions^b^	Factors associated with accuracy
Percentages of agreement, where the lowest percentage concordance is 33%, first quartile is 64%, median is 71%, third quartile is 75%, and highest is 100%^c^							
Sulmasy	1994	“Medical Directive” of Emanuel and Emanuel case vignettes^d^	5, “Brain disease”	Surrogate Accuracy in Matching Patient Preferences Scale (SAMPPS), kappa coefficients, and multiple linear regression analysis	50 (*n* = 100)	Surrogate accuracy ranged from 57% to 81% across scenarios, with an average of 67% accuracy overall	Prior discussion of preferences was significantly associated with a 29% increase in surrogate accuracy on average. Nonchurch-going behavior was significantly associated with a 34% increase in surrogate accuracy on average.
Matheis-Kraft^q^	1997	Preferences for life-sustaining treatment questionnaire^e^	1, “Permanent confusion”	Percentages of agreement and kappa coefficients	60 (*n* = 120)	Surrogate accuracy ranged from 17%–97% across scenarios, with an average of 63% accuracy overall	Surrogates had higher accuracy after listening to patient discuss values with study team member compared to surrogates who did not listen to a discussion of patient values.
Sulmasy	1998	Author’s case vignettes	5, “Brain disease”	SAMPPS, chi-square or Fisher exact test, two-tailed *t*-tests, ANOVA, McNemar tests, Cochrane *Q* tests, and kappa coefficients	300 (*n* = 600)	Surrogate accuracy ranged from 37% to 73% across scenarios, with an average 66% accuracy overall	Odds ratios (95% confidence) were reported for factors increasing surrogate accuracy. Patient has spoken about end-of-life care in detail with surrogate: 1.89 (1.56–2.28) Patient has high school diploma: 1.73 (1.38–2.19) Surrogate has high school diploma: 1.50 (1.16–1.92) Surrogate does not go to church or temple: 1.48 (1.12–1.96) Patient has private insurance: 1.35 (1.06–1.73) Patient thinks that he or she will live for more than 10 years: 0.62 (0.51–0.75) Surrogate has experience with life-sustaining treatments: 0.41 (0.30–0.55)
Smucker^q^	2000	Life Support Preferences/Predictions Questionnaire^f^	7, Alzheimer’s disease	Percentage agreement and repeated-measures ANOVA	401 (*n* = 802)	Surrogates’ accuracy ranged from 74% to 99% across scenarios; the average accuracy rate was 74%–75%	None reported
Moorman	2008	1999 Detroit Area Study module “Health Care and End-of-Life Decisions case vignettes	1, Cognitive impairment	Multinomial logistic regression	2,750 (*n* = 5,500)	Surrogates made errors in 12%–22% of cases and uncertain in 11%–16% of cases, resulting in a range of accuracy from 62%–77% across scenarios	Patients having a discussion of preferences with another was associated with lower surrogate uncertainty, but no level or type of ACP was associated with decreased odds of surrogate error.
Zettel-Watson	2008	Life Support Preferences/Predictions Questionnaire^f^	1, Alzheimer’s disease	Author’s accuracy index, ANOVA, and kappa coefficients	249 (*n* = 498)	Husband accuracy ranged from 2% to 95% across disease scenarios, with an overall accuracy of 69% (*SD* 17%). Wife accuracy ranged from 1% to 97% across disease scenarios, with an overall accuracy of 74% (*S*D 15%).	Wives were significantly more accurate than husbands overall (*p* = .03) and in the Alzheimer’s disease scenario, where husbands’ accuracy was 59% (*SD* 36%) and wives’ accuracy was 70% (*SD* 34%).
Moorman	2009	1999 Detroit Area Study module “Health Care and End-of-Life Decisions case vignettes	2, Cognitive impairment	Structural equation modeling, maximum likelihood estimation, and asymptotic covariance matrix	2,750 (*n* = 5,500)	In the cognitive impairment scenario, 87% of surrogates made accurate predictions, compared to 74% in the pain scenario	Surrogates who had completed DPAHC or informal ACP conversations did not make more accurate predictions than other surrogates.
Volandes^q^	2009	Author’s structured questionnaire	1, Alzheimer’s or another dementia	Percentages of agreement, Chi-square tests, and *t-*tests	14 (*n* = 28)	In the experimental group, 100% of surrogates made accurate predictions, while 33% of surrogates made accurate predictions in the control group	An experimental group of surrogates shown a video depicting a patient with advanced dementia had much higher accuracy compared to surrogates only given a verbal description of the disease.
Whitlatch	2009	Values and Preferences Scale^g^	1, Cognitive impairment	Exploratory factor analysis and *t*-tests	267 (*n* = 534)	Surrogates accurately predicted the importance of 33% of values	Surrogates were generally accurate in predicting which values were important to patients but significantly underestimated the importance of those values in 16 of the 24 items (67%).
Chau	2010	Author’s structured questionnaire	1, Cognitive impairment	Univariate logistic regression	707 (*n* = 1,412)^n^	Surrogates to patients with CI were 62.5% accurate in predicting the patient’s preference for residential care, compared to 79.5% of surrogates to patients without CI. This difference is significant (*p* < .001).	None reported
Ayalon	2012	Author’s case vignettes	2, Alzheimer’s or another dementia	Bivariate analysis	53 (*n* = 106)	Surrogate accuracy in the dementia scenario was 68% for a feeding tube and 72% for CPR	None reported
Dening	2016	Life Support Preferences/Predictions Questionnaire^f^	3, Dementia	Multiple linear regression analysis	60 (*n* = 120)	Surrogate accuracy ranged from 20% to 71%, with an average of 39% accuracy	Surrogates were most accurate for the current state of health (average 51% accuracy) compared to severe stroke and coma (36%) and cancer (30%).
Bravo	2017	Author’s case vignettes	3, Severe dementia	Linear mixed models analysis	235 (*n* = 470)	Surrogates were 71% accurate for predictions in the state of severe dementia, compared to 36% accurate for stroke	Difference in quality-of-life ratings was a significant predictor of inaccuracy across all three hypothetical disease states (*p* < .001).
Fried	2017	Author’s structured questionnaire	3, Cognitive disability with memory loss	Mantel-Haenszel test for trend	350 (*n* = 700)	Surrogates agreed with patients about ACP participation in 66%–82% of ACP activities, with an average rate of agreement of 75%. Self-reported surrogate knowledge of patient preferences ranged from 15% to 30% across disease states.	More surrogates had knowledge of patient preferences across all three health states when both surrogate and patient agreed that communication about quantity vs. quality of life had taken place (30%) compared to agreement on having end-of-life conversations about life-sustaining (24%) or a healthcare proxy assignment (24%).
Bravo^q^	2018	Author’s case vignettes	1, Alzheimer’s or another dementia	Percentages of agreement and polychoric correlation coefficients	157 (*n* = 314)	Surrogate accuracy ranged from 43% to 83% across disease scenarios	None reported
Miller	2019	Care Values Scale^h^	1, Alzheimer’s or another dementia	Multilevel modeling and latent class mixture modeling	228 (*n* = 456)	75% of surrogates had a “shared understanding” of care values with the patient, while 25% of surrogates underestimated the importance of values to the patient	Surrogates significantly (*p* < .001) underestimated the importance to patients of who helps with care, of not being a burden, and of engaging in social activities.
Song^q^	2019	Author’s goals-of-care tool	1, Alzheimer’s or another dementia	Differences in pre-post scores and Kruskal–Wallis tests	23 (*n* = 46)	73.9% of surrogates accurately predicted patient preferences	None reported
Towsley^q^	2022	Author’s interview questions	1, Alzheimer’s or another dementia	Multilevel linear regressions and an additive model	36 (*n* = 124)^o^	Surrogate accuracy in the early intervention group was 58% at time of treatment (standard error [*SE*] 4.1%), 66% in the waitlist group at time of treatment (*SE* 5.3%), and 43% in the control group (*SE* 4.2%).	None reported
Mean difference in scores, where zero represents perfect concordance and greater distance from zero represents less concordance							
Schmid	2010	Illness Experience and Advance Planning Form^g^ and Life Support Preferences/Predictions Questionnaire^f^	5, Alzheimer’s disease	Chi-square tests, Mann–Whitney test, *t*-tests, raw difference of scores, and ANOVA	64 (*n* = 128)	Mean raw differences in scores ranged from −0.66 (*SD* 1.81) to 1.18 (*SD* 2.08).	Lower levels of ACP (completion of a living will, a DPAHC, and conversations with family about end-of-life care) predict undertreatment errors among Black surrogates and overtreatment errors in White surrogates. Higher levels of ACP increase surrogate accuracy across all races.
Parks	2011	Life Support Preferences/Predictions Questionnaire^f^	7, Alzheimer’s disease	ANCOVA and least significant difference tests	202 (*n* = 404)	Mean difference in scores was lowest for spousal surrogates at 0.10 (*SD* 0.94), and highest for adult children surrogates at 0.56 (*SD* 1.0). Other proxies had a mean difference in scores of 0.46 (*SD* 1.13).	Spouse surrogates were significantly more accurate than adult children (*p* = .007) or other surrogates (*p* = .085); surrogates in families with low conflict were more accurate (*p* = .036)
Scales where zero represents perfect concordance and greater distance from zero represents less concordance							
Reamy	2011	Values and Preferences Scale^h^	1, Alzheimer’s or another dementia	Multilevel modeling	266 (*n* = 532)	Low to moderate concordance. Incongruence score of 0.34–0.84 (range −1, 1)	Surrogates underestimated the importance of care values for patients on all five values measured.
Miller	2018	Care Values Scale^k^	1, Alzheimer’s or another dementia	Multilevel modeling	42 (*n* = 84)	Moderate concordance. Incongruence score of −0.49 to −0.21 (range −1, 1)	Surrogates underestimated the importance of care values for all four subscales measured.
Miscellaneous scoring of concordance							
Carpenter	2007	Brief Treatment Planning Questionnaire^m^	1, Alzheimer’s or another dementia	Intraclass correlation coefficients	64 (*n* = 128)	Moderate to good concordance. ICC = 0.36–0.66	None reported
Reamy	2013	Values and Preferences Scale^f^	1, Alzheimer’s or another dementia	Growth curve analysis and multilevel modeling	198 (*n* = 396)	Small annual reduction in concordance over disease progression. Concordance dropped by 2.76 points per year (range 50, 110)	None reported
Level of concordance not given or not applicable							
Turan	2011	Life Support Preferences/Predictions Questionnaire^e^	5, Alzheimer’s disease	Regression analysis	81 (*n* = 162)	Not given	Surrogates with secure attachment style were more accurate.
Kwak	2016	Author’s structured questionnaire	1, Alzheimer’s or another dementia	Multiple regressions and Pearson’s correlation coefficients	N/A (*n* = 141)^p^	Not applicable	Surrogates who did not know individual treatment preferences reported greater understanding of patient values (Beta = −0.19, *p* < .05) compared to surrogates who knew some or all treatment preferences.

*Notes*: This table uses “surrogate” and “patient” rather than the terms used in the remainder of the paper—“caregiver” or “care partner” and “care recipient” to reflect the terminology used in the literature itself. Additionally, not all surrogates studied in the articles above were the primary caregiver for the patient; therefore, use of the term surrogate or surrogate decision maker is more accurate. ACP, advance care planning; ANCOVA, analysis of covariance; ANOVA, analysis of variance; CI, cognitive impairment; DPAHC, durable power of attorney for health care.

^a^Listed is the measurement tool or scale for end-of-life treatment preferences or values only. Other scales given to participants of each study, such as the Mini-Mental State Exam or the Dyadic Relationship Scale, were not listed.

^b^For studies that included case vignettes with multiple disease trajectories (e.g., Alzheimer’s, heart failure, cancer, and frailty), surrogate accuracy for Alzheimer’s, dementia, or cognitive impairment was used.

^c^These numbers were produced by ordering every reported percentage of accuracy from lowest to highest and then taking the minimum, first quartile, median, third quartile, and maximum numbers. For studies that reported a range of accuracy rates, the average was used for ordering.

^d^ Emanuel and Emanuel (1989).

^e^ Cohen-Mansfield et al. (1991).

^f^ Beland and Froman (1995); Ditto et al. (2001).

^g^ Bookwala et al. (2001).

^h^ Whitlatch et al. (2005).

^k^This is Miller’s naming of the Values and Preferences Scale developed by Whitlatch et al. (2005, 2009).

^m^ Carpenter et al. (2007).

^n^“Excluding cases with complete information, a total of 707 elders and 705 caregivers were included in the analysis, with 475 pairs of elder-caregiver dyads” (Chau et al., 2010).

^o^This is a nondyadic study. Participants included nursing home residents (*n* = 36), their family members (*n* = 50), and staff members (*n* = 38).

^p^This is a nondyadic study. Participants were 141 designated healthcare proxies only.

^q^Indicates the study included an intervention.

This body of literature shows surrogates’ median accuracy rate is 71%. About half of the studies reported surrogates’ accuracy rate between 64% and 75%, while a quarter each reported surrogates were less than 64% accurate and more than 75% accurate. One study found a surrogate accuracy rate as high as 100%, but this was an intervention with only eight dyads in the experimental group (Volandes et al., 2009) and only measured three paths of care, making it simpler for surrogates to achieve a high accuracy rate compared with other studies that used more disease scenarios and treatment options. Another study found that surrogate accuracy was as high as 99% (Smucker 2000), but this was only for treatment in the “current health” state. This scoping review yielded no literature evaluating whether surrogates’ median accuracy is “high” or “low” at 71%. Identifying articles that measure surrogate decision makers’ ability to predict their care recipient preferences informs our analysis by providing concrete information on how often dementia care dyads reach ACP concordance. Forming and operationalizing a definition of care dyad ACP concordance would be of little value if most dementia care dyads were achieving ACP concordance already. However, because dementia care dyads vary so widely in their accuracy rates, an operationalized definition will lay the foundation for future study on how to measure and improve care dyad ACP concordance.

#### Objective 3: Ascertain best practices facilitating care dyad ACP concordance

Participation in ACP was found to improve the accuracy of surrogate decision makers’ predictions in five of the articles included in the scoping review. Surrogate decision makers who listen to the care recipient discuss their values, engage in a discussion with the care recipient regarding their ACP preferences, or are involved with more than one type of the three foundational steps of ACP—completion of an advance directive, a durable medical power of attorney, and informal conversations about care preferences—demonstrate improved surrogate accuracy (Fried et al., 2017; Matheis-Kraft & Roberto, 1997; Schmid et al., 2010; Sulmasy et al., 1994, 1998) and reduced surrogate uncertainty (Moorman & Carr, 2008). Relational and gender factors were also reported in the literature as contributing to surrogate decision-maker accuracy. Turan et al. (2011) found that surrogate decision makers with a secure attachment style were more able to correctly predict the ACP wishes of the care recipient; secure attachment may therefore contribute to care dyad ACP concordance. Similarly, Miller et al. (2018) found that more positive interactions and less relationship strain were associated with higher surrogate accuracy, and therefore may foster care dyad ACP concordance. Two additional articles studied the impact of gender and relationship type on surrogate accuracy: Zettel-Watson et al. (2008) found that wives made more accurate predictions than husbands, and Parks et al. (2011) found that spouses were more accurate than children or other surrogates.

Six articles provided guides for completing ACP for a person with dementia. These were theoretical articles that provided information on the process of ACP and how to maximize the amount of knowledge transferred from the care recipient to the care partner. Table 3, which summarizes these articles, shows that none of these guides emphasized reaching a point of mutual understanding among care dyads. Even these best practice guides for ACP in dementia do not incorporate the concept of care dyad ACP concordance. However, these guides still provide useful information on how to maximize the transfer of knowledge within a dementia care dyad. Knowledge transfer from the care recipient to the surrogate decision maker is critical for fostering care dyad ACP concordance, as for surrogate decision makers to understand and commit to following through with the care recipient’s wishes, they must first know these wishes.

**Table 3.** Articles Studying the Process of ACP in the Context of Dementia

First author	Year	Intervention	Key ACP steps
Bosisio	2018	Yes	• Choose the right moment to initiate ACP • Adapt tools to patients’ cognitive ability • Designate a person responsible for managing ACP decisions
Dassel	2019	Yes	• Engage in ACP early in the illness trajectory • Use a values-based rather than treatment-based approach • Repeat conversations as illness progresses • Identify a surrogate decision maker • Use a structured tool designed for dementia to record preferences • Share recorded preferences with surrogate, family, and medical providers
deLima Thomas	2018	No	• Engage in ACP early in the illness trajectory • Use a values-based rather than treatment-based approach • Repeat conversations as illness progresses • Assess patient’s cognitive ability to participate in decision making • Identify a surrogate decision maker • Use a structured tool designed for dementia to record preferences • Document preferences in the electronic health record
Macchi	2022	No	*Mild/early-stage dementia* • Engage in ACP early in the illness trajectory • Integrate ACP into daily care • Recognize “triggers” for ACP: diagnosis, changes in health status, rapid cognitive decline • Repeat ACP annually or at a “trigger” point • Introduce ACP alongside other future planning, such as financial planning • Identify and address barriers, concerns, and misconceptions about ACP *Moderate stage dementia* • Assess patient’s cognitive ability to participate in decision making • Discuss life-sustaining treatment preferences *Advanced stage dementia* • Discuss preferences for hospitalization, hospice, and specific interventions (artificial feeding and hydration, antibiotics)
Mezey	2000	No	• Identify the best time of day and environment for conversation • Have a trusted person present for the conversation • Take ethnicity and culture into consideration • Suggested documents include an advance directive, healthcare power of attorney, values history, POLST, and do not hospitalize order • Consider advance instructions for broad, rather than specific, types of care • Help patients articulate under what conditions hospitalization and comfort care should be pursued
Whitlatch	2013	No	• Balance best interests with preferences • Maintain the decision-making involvement of the care recipient as long as possible • Determine care values for the person with dementia • Determine preferences for who will provide care to the care recipient

*Notes*: ACP, advance care planning; POLST, Physician Orders for Life-Sustaining Treatment.

Four articles directly suggested appointing a surrogate decision maker (Dassel et al., 2019; deLima Thomas et al., 2018; Mezey et al., 2000) or designating a person “responsible for managing ACP” (Bosisio et al., 2018). These articles suggested that surrogates should initiate the process of ACP early in the dementia illness trajectory so that planning takes place when the person with dementia is most able to participate alongside the surrogate (Dassel et al., 2019; deLima Thomas et al., 2018; Macchi & Lum, 2022). Two more indicated choosing a time and place appropriate for ACP discussion (Bosisio et al., 2018; Mezey et al., 2000).

Four of these six articles also recommended eliciting care recipient values. Two suggested that ACP should primarily focus on a discussion of the patient’s care values to create a more flexible set of guidelines for the surrogate decision maker (Dassel et al., 2019; deLima Thomas et al., 2018). Whitlatch (2013) also recommended including care values as part of the discussion of treatment preferences but does not advocate making it the primary approach. Similarly, Mezey et al. (2000) recommended the completion of a Values History form, which is a more formalized way to elicit care recipient values.

Three articles recommended that care partners conduct a professional evaluation of the care recipient’s cognitive ability as changes to care plans are made over the course of the disease (Bosisio et al., 2018; deLima Thomas et al., 2018; Macchi & Lum, 2022). Three articles indicated the importance of repeating ACP conversations as the illness progresses (Dassel et al., 2019; deLima Thomas et al., 2018; Macchi & Lum, 2022). Macchi and Lum’s (2022) suggestion to repeat conversations annually or whenever the care recipient has a noticeable change in either health status or cognitive functioning is particularly compelling. Finally, three articles suggested using a structured ACP tool (Dassel et al., 2019; deLima Thomas et al., 2018; Mezey et al., 2000) so that the care partner is not relying solely on their memory to enact the care recipient’s values and preferences. Taken together, these articles state that appointing a trusted and capable surrogate decision maker, beginning the ACP process as early as possible in the dementia disease trajectory, eliciting patient values as well as preferences, and repeating ACP conversations throughout the disease course are critical factors in best practices for completing ACP in the context of dementia. These crucial steps add to our knowledge of how to foster care dyad ACP concordance.

Of the 34 articles included in this scoping review, only two (5.8%) directly addressed the process of achieving care dyad ACP concordance (Black et al., 2009; de Vries & Drury-Ruddlesden, 2019) and both were qualitative studies. Black et al. (2009) examined “how surrogate decision makers for dementia patients developed an understanding of patient preferences about end-of-life (EOL) care and patient wishes” (p. 627). They developed a three-part model of surrogate understanding: facilitators and barriers to ACP, methods of eliciting care recipient preferences, and the surrogate’s final understanding of care recipient preferences. Factors that increased the chance of care recipients expressing their preferences were (a) encouragement from others, (b) the care recipient’s decline in health, (c) the death or illness of others, and (d) and being “dispositionally inclined to plan” (p. 635). Barriers to care recipients expressing their wishes were (a) not being inclined to discuss or plan, (b) not having thought about it, (c) deferring the decisions to others, and (d) arriving at the decision too late due to cognitive impairment. Second, surrogates had three primary methods of eliciting care recipient preferences: an advance care directive, informal discussions, and “other ways of knowing,” which included knowing the person’s values, health decisions made in other contexts, and information learned through discussions with friends. The third part of the model entailed the surrogate’s final understanding of the care recipient’s preferences. Surrogates that reported having previous discussions about end-of-life care and an advance directive from their care recipient were able to describe more specific preferences than surrogates who had only one or neither of these two types of ACP.

The other article directly addressing care dyad ACP concordance was authored by de Vries and Drury-Ruddlesden (2019). The investigators conducted interviews with 23 care partners of people with dementia to describe “how informal conversations and discussions within the family relating to preferences at the end of life” (p. 3021) that occurred over many years provided surrogates with knowledge of the care recipient’s preferences. The key finding of this study was that dyads reached concordance through many “ordinary everyday conversations” (de Vries & Drury-Ruddlesden 2019, p. 3021). This finding indicates care dyad ACP concordance can be fostered through an open dialogue about end-of-life care values and preferences that are revisited over the life course.

### Scoping Review Limitations

This scoping review was unique, as over 90% of the articles screened were found via citation chaining rather than database searching. Although this made it possible to find a broad range of studies, we cannot be certain that we included all relevant articles. There is a possibility that relevant articles outside the “chain” of citations were missed by both the citation chaining and the database search of articles.

### Scoping Review Discussion and Implications

In conclusion, this scoping review aimed to uncover a definition, identify measures, and understand how to achieve care dyad ACP concordance. Personal attributes, relationship factors, and prior engagement in both formal and informal ACP were identified as factors contributing to surrogate decision makers’ improved accuracy in predicting the ACP wishes of the care recipient, which may contribute to care dyad ACP concordance. Some of these factors are modifiable (completing ACP documentation and having informal ACP discussions; Fried et al., 2017; Matheis-Kraft & Roberto, 1997; Schmid et al., 2010; Sulmasy et al., 1994, 1998), whereas other factors such as gender and type of relationship are not (Miller et al., 2018; Parks et al., 2011; Turan et al., 2011; Zettel-Watson et al., 2008). A greater understanding of how to engage in the process of care dyad ACP concordance and how to achieve ACP concordance is crucial to both members of the dyad to prevent unnecessary and unwanted medical treatments for the care recipient and negative psychological outcomes for the surrogate decision maker (Anderson et al., 2009; Azoulay et al., 2005; Fetherstonhaugh et al., 2017, 2019; Makino et al., 2020; Torke et al., 2014; Wilkins, 2021).

Our results also illuminated the fact that a definition of care dyad ACP concordance was not found in the articles identified through the scoping review. A formal definition of ACP concordance lays the foundation for future work creating a clinical measure of care dyad ACP concordance that healthcare teams can use to guide care dyads through the ACP process after a dementia diagnosis.

## Part 2: Qualitative Analysis

### Qualitative Analysis Background and Objectives

#### Objectives

The primary objective of this qualitative analysis was to identify any common themes embedded in dementia care dyad ACP conversations that foster care dyad ACP concordance. The secondary objective was to use these findings to aid in forming a definition of care dyad ACP concordance.

#### Background

The LEAD Study (*Dassel et al., 2019), an intervention designed to facilitate ACP in the context of dementia, was based on the LEAD Guide, an end-of-life care planning tool that helps people express their values and preferences for end-of-life care. First, the LEAD Guide assesses three value domains: quality of life, burden, and decision making. Second, the Guide elicits preferences for location of care, life-prolonging measures, and controlling the timing of death in the current state of health and in a future state of severe dementia. It also includes an open-ended response section to describe any other end-of-life values or preferences.

The LEAD Study was comprised of three modules. Module 1 asked the care recipient to complete the LEAD Guide with their own values and preferences, whereas the care partner completed the LEAD Guide with predictions of the care recipient’s values and preferences. This prepared the dyad for Module 2: a dyadic conversation about the care recipient’s end-of-life values and preferences, with the goal of transferring knowledge to the care partner. Within this conversation, the dyad was instructed to compare the care partner’s predictions of the care recipient’s end-of-life values and preferences to the care recipient’s responses. This allowed the dyad to identify areas in which they were nonconcordant. Each dyad had the option of recording this conversation, and those who agreed received a Zoom link and recording instructions via email. These recorded conversations provided the data relevant to this paper and the dyadic outcome of concordance. Module 3 helped participants share their LEAD Guide with their health providers, family, and friends. The full study, including the recording and analysis of the ACP conversations used here, was approved by the University of Utah Institutional Review Board [00162125].

### Qualitative Analysis Research Design and Methods

#### Qualitative analysis sample and setting

Dyads included one care partner and one care recipient. Dyads were eligible to participate in the LEAD Study if (a) care recipients were aged 50+ and showed at least one of the 10 Alzheimer’s Association “warning signs” of dementia, and (b) the care partner was aged 18+ and identified as the spouse, long-term partner, or adult child of the care recipient. Of the 61 eligible dyads, 7 completed in-depth recordings of their conversations from Module 2. The recorded conversations varied in length, with a time range of 2 min 50 s minimum to 24 min 48 s maximum, and a median time of 13 min 41 s. Five of the conversations included both video and audio recording, and two conversations were audio recording only.

Six of these seven dyads who recorded conversations had a spousal relationship, whereas the seventh was that of parent/child. Care partners ranged in age from 48 to 84; five were White non-Hispanic, one was White and Hispanic, and one was Native Hawaiian. Three care partners completed some college or technical school, two completed college, and two had postgraduate degrees. Care recipients ranged in age from 55 to 84; five were White non-Hispanic, one was White and Hispanic, and one was Native Hawaiian. One care recipient had completed some college or technical school, five had completed college, and one had completed a postgraduate degree.

#### Qualitative analysis methods

The conversations were conducted, recorded, and transcribed using the Zoom recording and transcription program. We evaluated the seven video recordings using Descriptive Qualitative Inquiry (Sandelowski, 2000, 2010). Qualitative descriptive inquiry permits researchers to remain “data-near” to the interview narratives in the naturalistic setting of care planning conversation. The data-near approach (Sandelowski, 2010) emphasizes accepting participant comments and dialogue at face value and fosters respect for the relationship between the care partner and care recipient as narrated.

Next, we reviewed transcripts while viewing each video and verified transcription accuracy. We performed an initial phase of line-by-line in vivo coding, followed by line-by-line process coding. Using both coding approaches, the data were coded at the level of phrases, so each line of text often had more than one code assigned, and multiple codes could be assigned to the same phrases. We then generated a list of structural codes (Saldana, 2021) to use in a primary phase of coding to categorize and organize segments of data by content and concept. The second author conducted this data checking through the level of phrase. Patterns in the phrases were discussed with the first, second, and third authors in an inductive approach, relating this to interactions observed in videos, with 95% initial agreement followed by adjudication to achieve consensus. This discussion produced themes that were verified by the entire research team.

### Qualitative Analysis Results

We discerned seven themes that all the ACP videos had in common. Themes included (a) respect/regard for each other as evidenced by courteous dialogue, (b) use of clarifying processes through the exchange of statements and questions, (c) exploring various health care scenarios to justify values and preferences, (d) affirmation of understanding by each member of the care dyad as evidenced by head nodding and statements of agreement, (e) recognizing uncertainty in unanticipated situations that may arise from future health changes, (f) humor and expression of positive emotions, such as smiling and sharing stories, and (g) trust, as evidenced by specific affirmation that the care recipient is certain that the care partner understands and will do their best to honor preferences. These themes are defined with examples in Table 4. Notably, all seven dyads who completed the recorded conversations voiced confidence that they had a high-quality, shared understanding of the care recipient’s end-of-life values and preferences.

**Table 4.** Themes Identified in Qualitative Analysis of Dementia Care Dyad Advance Care Planning Conversations

Theme	Definition	Example quote
Respect/regard	Courteous dialogue within the dyad	*Care Recipient:* “I said I am concerned about being an emotional burden on my family.” *Care Partner:* “Okay.” *Care Recipient:* “Just because of my intense emotions and sensitivity. I think the emotional is what is more difficult for people. Why did you say ‘only somewhat of a burden’?” *Care Partner:* “I don’t know why I said ‘somewhat’ … but I see … emotional … So (yes), I get that.”
Clarifying processes	Exchange of statements and questions	*Care Recipient:* “So for location of my care, I prefer home. But if it a super big burden (for you), then somewhere else, for all the work …” *Care Partner:* “And the stage of our life … if 60 years old or 80 years old (that matters)” *Care Recipient:* “If I have AD, ‘where I am’ … it is not necessarily my right to make that decision … I just want to be cared for by patient people.”
Conveying health care scenarios	Using possible care examples to justify values and preferences	*Care Recipient:* “Now … a breathing machine. If medical advice says ‘no hope for improvement’ … that’s a really different question from something that is a high probability of being temporarily so. But (with AD), no, absolutely not, if I added that dimension (of dementia).”
Affirmation of understanding	Voicing agreement of understanding	*Care Partner:* “So longevity of life for you …” *Care Recipient:* “Is not as important as quality of life.” *Care Partner:* “Yes, okay.”
Recognizing uncertainty	Agreeing that all possible events cannot be anticipated	*Care Partner:* “(It’s) the nitty-gritty-the question of quality of life … is a month too little, too long?” *Care Recipient:* “Weeks and months is too long, I mean during the initial incident in the beginning of that incident if I wasn’t able to be revived just by restarting my heart and it started beating on its own, then don’t leave me hooked up tomorrow.”
Humor/ expressed positive emotions	Being lightly amusing or cheerful to soften discussion	*Care Recipient:* “I don’t want to be no type of burden, period.” *Care Partner:* “So, if you don’t want to be a burden … you can’t say ‘neither agree or disagree.” *Laughs* *Care Recipient:* “Right, right. But if it is something just like for a week (that) I need help …” *Care Partner:* “… This is for long term stuff.” *Care Recipient: laughing quietly*. “Okay, yeah, I should have kept in mind this is talking about end-of-life.” *Smiles at Care Partner*
Trust	Conveying trust that decisions can be implemented	*Care Partner:* “If you were incapacitated, you want me to make that decision?” *Care Recipient:* “That would be fine.” *Care Partner:* “I was just curious. I never really thought about it.” *Care Recipient:* “If you abide by my wishes.” *Laughs* Care Partner: “I wouldn’t say I would do it, if I wasn’t going to be abiding by your wishes … (and) if I didn’t agree with what you were saying.” *Smile at Care Recipient*

*Notes*: AD, Alzheimer’s disease.

Each of the seven dyads showed evidence of all thematic elements during the interview, yet each conversation was unique. Four care partners voiced surprise at care recipient answers, which gave care recipients the opportunity to transfer knowledge of their values and preferences to their care partner. These same four dyads went back and forth with specific healthcare examples, such as describing what kind of care they would want with a certain disease trajectory, or the conditions that would lead to them wanting to be placed in a nursing home. All dyads recognized they could not anticipate every healthcare scenario. In discussing the uncertainty surrounding the use of artificial life support, one care partner asked, “(it’s) the nitty-gritty-the question of quality of life … is a month too little, too long?” Their care recipient clarified, “weeks and months is too long, I mean during the initial incident in the beginning of that incident if I wasn’t able to be revived just by restarting my heart and it started beating on its own, then don’t leave me hooked up tomorrow.” Six care partners voiced that deeper understanding of the undergirding values behind preferences could be useful if faced with an unexpected decision. Of the five for which we had video recording, we saw dyads smiling at each other; all dyads shared humorous comments and/or stories to reduce tension that the discussion might have elicited. All seven dyads conveyed a feeling of mutual respect, even when the care partner was initially surprised or disagreed with the care recipient’s position on an issue. Finally, all care recipients shared statements of trust about the care partner’s willingness to honor the care recipient’s goals and wishes, even if it was not the expressed preference of the care partner for the care recipient.

### Qualitative Analysis Limitations

The analysis was limited to the seven fully video-recorded interviews in this pilot phase, and as such, results may not be generalizable beyond similar groups. Although we observed all elements in all dyads, we cannot confidently claim data saturation. Second, we report no negative findings of inability to achieve concordance. Negative findings in this research setting would have been helpful to identify and describe those care dyads unable to achieve care dyad ACP concordance on their own. This will be an important consideration in future investigations. Finally, we recognize that a selection bias exists where dyads may have been more likely to enroll in the study if they were already interested in having these conversations, had better communication skills, and or had stronger dyadic relationships. Additionally, not all dyads enrolled in the study chose to participate in the conversation recordings; this indicates there may be a further selection bias at play, and that only dyads who were the most prepared for an ACP conversation chose to record their conversations. Dementia care dyads who did not opt in to recording their conversations may therefore represent dyads who would have less success in achieving ACP concordance.

### Qualitative Analysis Discussion and Implications

The qualitative analysis provided seven examples of care dyad ACP conversations that result in ACP concordance. The dyads also followed a format in determining their original level of concordance, prior to the conversation in which a surrogate decision maker predicted the end-of-life care values and preferences of the care recipient, and then the accuracy of those predictions was reviewed. This was very similar to many studies included in the scoping review, which found that many surrogate decision makers do not have a 100% rate of accuracy in predicting their care recipient’s values and preferences. This result from the scoping review was also present in the qualitative analysis, as some care partners expressed surprise at their care recipient’s value and preference responses.

Statements from care recipients that they trusted their care partner to carry out their wishes were the most critical contribution of the qualitative analysis to our definition of ACP concordance. Although our original concept of concordance focused primarily on the transfer of knowledge from care recipients to their care partners, this element of the recorded conversations contributed to the final key component of our definition, which is the willingness of the care partner to carry out their care recipients end-of-life care wishes—even if they do not like or agree with those wishes.

Because these dyads were able to achieve a high level of ACP concordance, we believe the common themes identified from their video recordings shed light onto the process of achieving ACP concordance. Although further study is needed to determine which themes are the most critical, these findings are a starting point for examining ACP conversations that result in ACP concordance. Further research on this topic is needed to develop a greater understanding of dyads that struggle to achieve concordance. An operational definition of care dyad ACP concordance can aid social workers, hospice nurses, and other clinicians in identifying and providing clinical aid to these care dyads.

## Discussion and Implications

Although clinical practice may assume concordance is the key outcome of ACP, there remains a theoretical gap in the literature on how to operationalize care dyad ACP concordance. To address this gap in the literature, we conducted a scoping review and qualitative descriptive analysis of dyadic data from a pilot study and used the findings to develop the following working definition of care dyad ACP concordance. Care dyad ACP concordance is *when care recipients and care partners both understand a care recipient’s end-of-life values, understand the end-of-life preferences informed by those values, and the care partner expresses a willingness to accomplish the care recipient’s wishes to the best of their ability.*

We believe defining ACP concordance will improve outcomes of the ACP process for dementia care dyads by advancing research measurement and through clinical application. We intend to use this definition to create a clinically usable instrument to assess care dyad ACP concordance. A validated instrument will allow us and other researchers to improve ACP intervention design and outcomes by measuring care dyad ACP concordance specifically and by separating it conceptually from goal-concordant care. In clinical settings, an ACP concordance instrument could help hospice and nursing home clinicians assess care dyads’ level of ACP concordance and identify those in need of additional support. When future research more clearly establishes the steps necessary to achieve care dyad ACP concordance, these steps can be shared with care dyads to help guide their process. We believe intentional inclusion of ACP concordance in research and clinical settings will help care dyads have higher-quality ACP conversations that prepare surrogate decision makers for their role and increase goal-concordant care. Improving ACP concordance will enable dementia care partners to experience less strain, depression, anxiety, uncertainty, and trauma-related symptoms (Anderson et al., 2009; Azoulay et al., 2005; Fetherstonhaugh et al., 2017, 2019; Makino et al., 2020) and will help care recipients have fewer undesired healthcare outcomes (Torke et al., 2014; Wilkins, 2021).

## Supplementary Material

gnae029_suppl_Supplementary_Materials

## Data Availability

The de-identified transcripts, analysis, and results of the qualitative analysis are available by request. Raw data are in video format, and therefore cannot be de-identified and shared. A full list of screened articles from the scoping review is also available upon request. The scoping review was not registered, as PROSPERO does not accept scoping reviews for registration.
